# H3K27ac chromatin acetylation and gene expression analysis reveal sex- and situs-related differences in developing chicken gonads

**DOI:** 10.1186/s13293-022-00415-5

**Published:** 2022-02-08

**Authors:** Yunqi Jiang, Zhelun Peng, Qiu Man, Sheng Wang, Xiaochen Huang, Lu Meng, Heng Wang, Guiyu Zhu

**Affiliations:** 1 Department of Animal Genetics, Breeding and Reproduction, College of Animal Science and Technology, Shandong Agricultural University, Taian, China; 2 Key Laboratory of Agricultural Animal Genetics, Breeding and Reproduction of Ministry of Education, College of Animal Sciences and Technology, Huazhong Agricultural University, Wuhan, China

**Keywords:** Chicken, Gonad, Sex differentiation, Asymmetric differentiation, Chromatin, H3K27ac

## Abstract

**Background:**

Birds exhibit a unique asymmetry in terms of gonad development. The female left gonad generates a functional ovary, whereas the right gonad regresses. In males, both left and right gonads would develop into testes. How is this left/right asymmetry established only in females but not in males remains unknown. The epigenetic regulation of gonadal developmental genes may contribute to this sex disparity. The modification of histone tails such as H3K27ac is tightly coupled to chromatin activation and gene expression. To explore whether H3K27ac marked chromatin activation is involved in the asymmetric development of avian gonads, we probed genome-wide H3K27ac occupancy in left and right gonads from both sexes and related chromatin activity profile to the expression of gonadal genes. Furthermore, we validated the effect of chromatin activity on asymmetric gonadal development by manipulating the chromatin histone acetylation levels.

**Methods:**

The undifferentiated gonads from both sides of each sex were collected and subjected to RNA-Seq and H3K27ac ChIP-Seq experiments. Integrated analysis of gene expression and active chromatin regions were performed to identify the sex- and situs-specific regulation and expression of gonadal genes. The histone deacetylase inhibitor trichostatin A (TSA) was applied to the undifferentiated female right gonads to assess the effect of chromatin activation on gonadal gene expression and cell proliferation.

**Results:**

Even before sex differentiation, the gonads already show divergent gene expression between different sexes and between left/right sides in females. The sex-specific H3K27ac chromatin distributions coincide with the higher expression of male/female specification genes in each sex. Unexpectedly, the H3K27ac marked chromatin activation show a dramatic difference between left and right gonads in both sexes, although the left/right asymmetric gonadal development was observed only in females but not in males. In females, the side-specific H3K27ac occupancy instructs the differential expression of developmental genes between the pair of gonads and contributes to the development of left but not right gonad. However, in males, the left/right discrepancy of H3K27ac chromatin distribution does not drive the side-biased gene expression or gonad development. The TSA-induced retention of chromatin acetylation causes up-regulation of ovarian developmental genes and increases cell proliferation in the female right gonad.

**Conclusions:**

We revealed that left/right asymmetry in H3K27ac marked chromatin activation exists in both sexes, but this discrepancy gives rise to asymmetric gonadal development only in females. Other mechanisms overriding the chromatin activation would control the symmetric development of male gonads in chicken.

**Supplementary Information:**

The online version contains supplementary material available at 10.1186/s13293-022-00415-5.

## Highlights

- Sex-specific H3K27ac modifications in gonads were strongly associated with sex-biased expression of sex-determining genes.
- Both male and female gonads show left-biased H3K27ac deposition, but a positive correlation between H3K27ac marked chromatin activity and gene up-regulation was found in females but not males.
- Blocking histone de-acetylation in the female right regressing gonad could stimulate cell proliferation and ovarian gene expression.

## Introduction

The chick exhibits an unusual asymmetry in terms of the development of the gonads. Around E4.5 in chicken embryo (Hamburger Hamilton Stages 24, HH24), the morphological appearance of the gonads is very similar between males and females. At this stage, the gonads from both sexes were still in a "bipotential" state and had the ability to differentiate into either ovaries or testes [1]. As sex differentiation proceeds, the male and female gonads are distinctively developing both genetically and morphologically. Similar to mammals, male chicken embryos would develop bilateral gonads during sex determination and eventually form symmetrical testes. However, the undifferentiated gonads from female embryos would develop asymmetrically along the left/right axis, with the left gonad developing into a functional ovary but the right gonad regresses until degenerated [2–5]. These two types of observed asymmetries in gonadal development, namely the male/female asymmetry in sex differentiation and the left/right asymmetry in ovary formation in females, have been partially elaborated by the transcriptome and gene perturbation analysis [6–8]. Very little is known about the role of epigenetic regulation in asymmetric gonadal development in chicken [9].

Studies on mammals showed that both genetic and epigenetic factors are the major players in sex determination and gonad development [10]. The Y chromosome derived *Sry* induces *Sox9* up-regulation in the early gonad is crucial for initiating testis development. The XX gonads lack *Sry* and commit the female gene expression program to develop into ovaries [11, 12]. The epigenetic regulation of chromatin activity was vital important to drive the sex differentiation. For instance, the mis-regulation of the activities of core enhancers upstream of *Sox9* caused downregulation of *Sox9* and complete male-to-female sex reversal in both mice [13] and human [14]. The analysis of gonadal chromatin landscape in mice also suggests that histone modifications contribute to the accurate gonadal gene expression in each sex and thus play an integral role in sex determination [15]. Therefore, the dynamics of chromatin activity must be tightly and precisely controlled to establish the accurate gene expression profile for gonadal sex differentiation.

The chicken embryonic gonad differentiation provides an excellent model for studying the functional relevance of chromatin activation during sex- and situs-specific tissue development. In this study, we performed ChIP-seq for H3K27ac, a histone acetylation modification indicative of active chromatin regions [16, 17], in undifferentiated bipotential gonads from both sides of each sex. The active chromatin regions and cis-regulatory-elements (CRE) adjacent to sex-determining and gonadal developmental genes were identified. We found the sex-specific H3K27ac chromatin modifications could correlate to the specific expression of sex differentiation genes in each sex, but the situs-specific H3K27ac chromatin modifications appear to only function in females. The current high-throughput chromatin analysis in chicken gonad will advance our understanding of the developmental asymmetricity in both male/female determination and left/right gonadal differentiation. We also anticipate to identify the sex-determining genes and the associated chromatin regions that can be targeted to genetically modulate sex differentiation in chickens [18], which is a goal of the poultry industry.

## Materials and methods

### Chicken embryo incubation and gonad collection

Fertilized eggs of White Leghorn chickens were incubated at 37 ℃ and 60% humidity until HH stage 24 (embryonic day 4.5, E4.5). The left and right gonads were detached from the mesonephros (primitive kidney) under a stereo microscope and cleaned in cold PBS. The isolated gonads were either snapped frozen in liquid nitrogen and stored at -80℃ for RNA-Seq and RT-qPCR, or dissociated into cell suspensions for ChIP. At the same time, a small piece of embryonic heads or other tissues were collected and stored at −20 ℃ separately for PCR sexing. All animal procedures were performed according to the protocols of the Huazhong Agricultural University and the Institutional Animal Care and Use Committee.

### Genetic sexing

For the genetic sexing of embryos, a small piece of tissue was digested to extract DNA with a genomic DNA extraction kit (Tiangen). Genetic sexing was carried out by a standard genotyping PCR protocol focused on the chicken *CHD1* (Chromo-helicase-DNA-Binding) gene. The primers are listed in Additional file 1: Table S1. These primers are targeting the introns of the *CHD1* gene, which is located on the Z (*CHD1*Z, 482 bp) and W-chromosome (*CHD1*W, 326 bp) [19]. PCR reactions were 95 ℃ for 5 min followed by 30 cycles of 95 ℃ for 30 s, 51℃ for 30 s, 72 ℃ for 30 s, and a final extension step of 72 ℃ for 10 min. The amplicons were separated from one band (Z) in the case of male or two bands (Z + W) in the case of female on agarose gel.

### Total RNA extraction and RNA-Seq

The gonads were pooled according to sex and side. Each biological replicate included 5 gonads. Total RNA was extracted with TRIzol reagent (Simgen, 5301100). High-throughput RNA sequencing was performed using a HiSeq X commercial service.

### Quantitative real-time PCR

Dissected gonads from embryonic stage HH24 were pooled according to sex and side. Each sample included more than three biological replicates and one replicate contained at least three gonads of the same type. Total RNA was isolated by using the TRIzol reagent. The cDNA was prepared using PrimeScript RT reagent Kit with gDNA Eraser (Takara, RR047A). Analysis of mRNA expression was performed with SYBR green fluorescent dye (ABclonal, RM21203). Quantitative PCR analysis was performed with ABclonal qPCR reagent and Bio-Rad CFX96/384 fluorescent quantitative PCR instrument. The PCR primer sequences are listed in Additional file 1: Table S1.

### RNA-Seq analysis

RNA-Seq data were quality checked with FastQC and removed low-quality sequences with trimmomatic (version 0.39). We used hisat2 (version 2.1.0) to gain read alignment by mapping reads against reference genome galGal6 (http://ftp.ensembl.org/pub/release-102/fasta/gallus_gallus/dna/) [20]. Gene expression quantification was performed with featureCount (version 2.1.0) and gene annotation document “Gallus_gallus.GRCg6a.102.chr.gtf” [21]. The gene information Query and Transformation were performed by ensemble bioMart database (http://asia.ensembl.org/biomart/martview/). The R programming language (version 4.0.3) and package DESeq2 (version 1.30.1) were adopted to differential expression analysis (|log2FC|≥ 1, *P* value < 0.05) [22]. Plots of PCA and heatmap were finished by R packages ggplot2 and heatmap. The GO (Gene Ontology) and KEGG (Kyoto Encyclopedia of Genes and Genomes) functional enrichment analysis were performed by the Metascape database (http://metascape.org/gp/index.html).

### Chromatin immunoprecipitation (ChIP)

For tissue ChIP, gonads were pooled by sex and side. Every sample included 2 biological replicates and each replicate contained at least ten gonads. A 30G syringe was used to produce a single cell suspension in cold PBS and then filtered through 40-μm strainer to remove cell clumps. Then the SSEA-1 antibody (Santa Cruz, sc-21702) conjugated magnetic beads were added into the cell suspension and incubated at room temperature for 15 min. The primordial germ cells were captured by the magnetic stands and the rest of gonadal cells were cross-linked using 1% formaldehyde at room temperature for 10 min followed by quenching using 200 mM glycine for 10 min at room temperature. The parameters of sonication were 34% power and 20 s ON/30 s OFF cycles for 6 min. Chromatin was incubated at 4℃ overnight with anti-histone H3-K27 acetylation antibody (Abcam, ab4729) at 15 ng/ul. After incubation, beads were washed three times with lysis buffer (0.1% SDS, 1% Triton X-100, 1 mM EDTA, 50 mM HEPES,150 mM NaCl) followed by two washes with high salt wash buffer (0.1% SDS, 1% Triton X-100, 1 mM EDTA, 50 mM HEPES, 350 mM NaCl) then one wash with LiCl wash buffer (0.25 M LiCl, 0.5% NP40, 0.5% sodium deoxycholate, 1 mM EDTA, 10 mM Tris–HCl pH 8.0) and finally two washes with TE pH 8.0 (10 mM Tris–HCl, 1 mM EDTA). Beads were then resuspended in 90 μl of freshly prepared ChIP elution buffer (1% SDS, 0.1 M NaHCO3) and incubated at 65℃ for 30 min with shaking [23]. IP and input samples were treated with Proteinase K (Ambion, AM2546) to reverse crosslinking. DNA was then extracted with phenol/chloroform/isoamyl alcohol, precipitated with 3 M sodium, and resuspended in 10 mM Tris–HCl. ChIP-Seq data were generated on Illumina HiSeq X Sequencer.

### ChIP-Seq analysis

The data quality control of ChIP-Seq data was performed same as RNA-seq. We used Bowtie2 (version 2.3.5.1) to gain read alignment by mapping reads against reference genome galGal6 (http://ftp.ensembl.org/pub/release-102/fasta/gallus_gallus/dna/) [24]. Peaks were called by MACS2 (version 2.1.1) with default parameters [25]. The R programming language (version 4.0.3) and package ChIPseeker (version 1.26.0) were adopted to perform peak annotation [22]. All alignment results were then converted to coverage bigwig files and normalized to the corresponding input using DeepTools (version 3.0.2) [26]. The bigwig formats can be visualized by software IGV (Integrative Genomics Viewer). To identify motifs significantly enriched for H3K27ac compared to flanking regions, HOMER (version v3.1) was used to apply the “findMotifsGenome” function [27]. And the “mergePeaks” function was used to do peak overlap in order to find the special and shared peaks among different samples.

### Injection of Trichostatin A and RT-qPCR analysis

Chicken embryos received an injection of 1 μl TSA (Trichostatin A) at 2.5 μg/μl or a vehicle DMSO as control. Eggs were injected at E4.5 on the right-side mesonephric region with a glass capillary needle and a PV830 Pneumatic PicoPump. One day later, the E5.5 gonads were removed and cleaned rapidly, then snap frozen in liquid nitrogen and stored at -80℃ individually for RNA extraction. Others were fixed in 4% paraformaldehyde overnight and incubated in 30% sucrose for 10 h at 4 °C for immunofluorescent staining. A piece of head tissue was collected and stored at -20℃ for determining the sex by PCR. At least three gonads were pooled together according to sex and side and considered as one biological replicate. Total RNA was extracted using TRIzol reagent (Simgen) and cDNA was prepared using PrimeScript RT reagent Kit with gDNA Eraser (Takara, RR047A). Analysis of mRNA expression was performed with SYBR green RT-PCR kit (ABclonal, RM21203). All primers are listed in Additional file 1: Table S1.

### Immunofluorescence staining and quantification

The gonads were fixed with 4% PFA and incubated with 30% sucrose and then dried on filter paper, and then soaked in Tissue Tek O.C.T. compound (Sakura Finetek Europe B.V.) before snap frozen in isopentane (2-methylbutane) cooled with liquid nitrogen. Sections were made at 5–10 µm thickness by Leica 1950 cryostat. The sections were fixed again in 4% PFA, permeabilized in 0.2% TritonX, blocked in 5% donkey serum. The antigen retrieval was performed in 10 mM pH 6.0 tri-sodium citrate (Sinopharm) at 75 °C for half an hour. The sections were incubated with the MCM2 antibody (Abcam, ab4461, 1:200) or H3K27ac antibody (Abcam, ab4729, 1:100) overnight at 4 °C and the corresponding secondary antibodies conjugated to Alexa Fluor 555 (Invitrogen, 1:500) for 2 h at room temperature. DAPI (50 μM) was used for the nuclear staining [28]. Five random representative images were taken from each gonad and MCM2^+^ or H3K27ac^+^ cells were counted.

### Statistical analysis

Kolmogorov–Smirnov test was used for comparison of the distribution of gene expression levels according to sexes or sides of gonads. Student’s *t* test was used for comparison of the differences of gene expression levels observed by qPCR. All analyses were performed using IBM SPSS Statistics Version 24 (SPSS Inc., Chicago, IL). All statistical tests were two-sided and *p* value < 0.05 was considered statistically significant (*p < 0.05, **p < 0.01, ***p < 0.001).

### Availability of data

Data generated have been deposited in NCBI database: PRJNA766745. The male and female gonad RNAseq data from previous reports include E4.5 and E6 RNA-seq: PRJNA171809 [29], E10, E12 and E14 RNA-seq: PRJEB26695 [30].

## Results

### The transcriptome analysis in different gonads of each sex

First, the gene expression profile of each type of gonads at E4.5 was investigated using transcriptome analysis. The rationale for using E4.5 (HH stage 24) is the onset of sex and situs specification from this stage onward. Of the clean reads from the RNA-seq, more than 94% were mapped to the chicken genome (galGal6) for each replication and more than 88% could be uniquely mapped to specific regions of chicken genome (Additional file 2: Table S2). The correlation between replications was more than 97% of each sample (Additional file 7: Fig. S1).

**Fig. 1:**
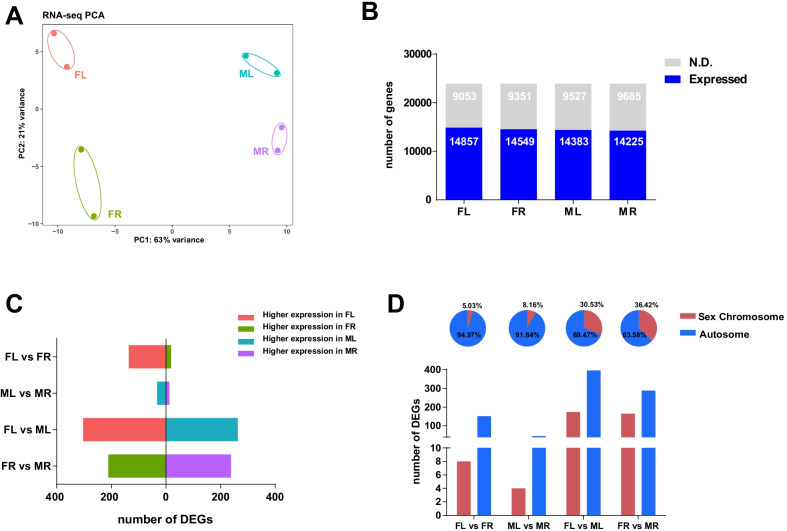
The transcriptome analysis of gonadal somatic cells from undifferentiated gonads of each sex. **A** Principal component analysis (PCA) of the transcriptomes of left and right gonads from males and females. Note the transcriptome from left and right gonads in females diverged more than that in males. FL: female left, FR: female right, ML: male left, MR: male right. **B** The total number of expressed genes (TPM ≥ 1) were similar in different gonads. **C** Number of differentially expressed genes (DEG) between different gonads. **D** The chromosome location of DEGs. The upper panel shows the proportions of DEGs located on sex chromosomes or autosomes. The bottom panel shows the numbers of DEGs located on sex chromosomes or autosomes. The situs-specific DEGs are more likely to locate on autosomes while the sex-specific DEGs are more likely to locate on sex chromosomes

The principal component analysis (PCA) was performed to gain insight into the relationships between different samples (Fig. 1A). The cluster analysis generated two groups based on sex, and then each group was further divided into left and right sub-groups, suggesting that the transcriptional discrepancy between females and males was greater than the difference between the left and right in each sex. Thus, for the gene expression patterns, the sex difference is greater than the situs difference. In total, 14,857, 14,549, 14,383 and 14,225 genes were detected in female left (FL), female right (FR), male left (ML) and male right (MR) gonad, respectively (transcripts per million (TPM) ≥ 1) (Fig. 1B). Thus, the number of expressed genes detected in right and left gonads in each sex at the early differentiation stage was almost equivalent.

Next, we identified the differentially expressed genes (DEGs) related to sex determination between FL vs ML and FR vs MR, and the DEGs associated with left/right differentiation between FL vs FR and ML vs MR. As shown in Fig. 1C, the numbers of sex-specific DEGs were much higher than the situs-specific DEGs, indicating that the sex differentiation may proceed ahead of left/right differentiation at this early stage of gonad development. We also noticed that there are much more DEGs between the FL vs FR than that of the ML vs MR, coincides with the PCA analysis in which the left/right separation between the two female gonads are more distant than the two male ones. Thus, the situs-specific gene expression patterns of the undifferentiated gonads already showed that the pair of gonads in females are more separated than that in the males.

When comparing left with right in each sex, more than 90% of the situs-specific DEGs were located on the autosomes and less than 10% of the situs-specific DEGs were located on the sex chromosomes. However, between different sexes, more than 30% of the sex-specific DEGs were located on the sex chromosomes (Fig. 1D). Hence, the sex chromosome genes are indeed more likely to be differentially expressed in different sexes, which is consistent with the previous report [31]. The subsequent function annotation analysis also suggested that these sex-biased genes could play important roles in sex differentiation.

### Functional annotation analysis of DEGs between “male vs. female” and “female left vs. female right” predict the gonad phenotype

**Fig. 2:**
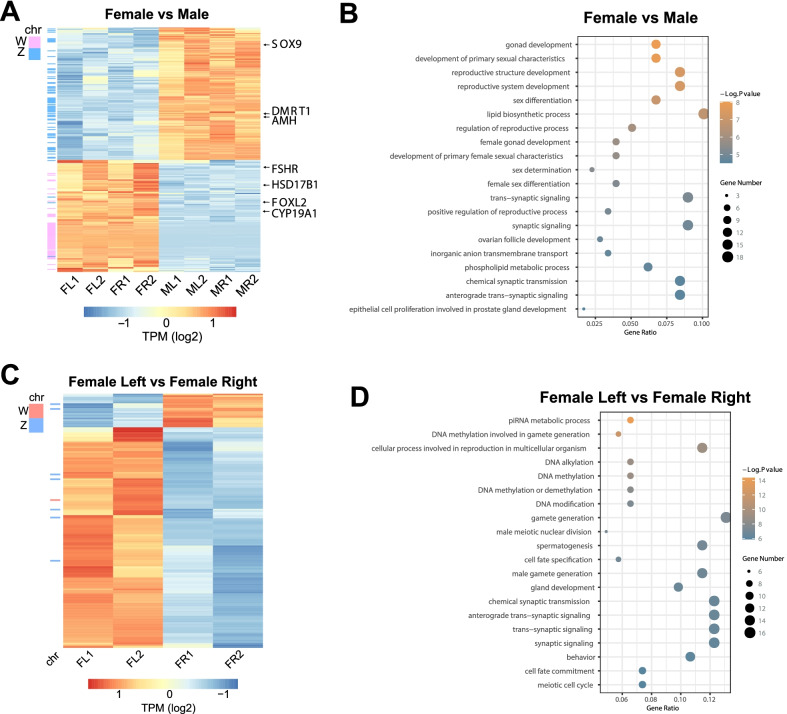
Functional prediction of the sex-biased and situs-biased DEGs. **A** The heatmap of the sex-specific DEGs for each sample. The red indicates the highest expression and dark blue is the least. On the left, the W-linked genes are indicated by pink lines while the Z-linked genes are indicated by blue lines. On the right, representative sex-biased genes were shown. **B** GO analysis of the sex-biased genes and the enriched GO terms were shown. **C** The heatmap of the situs-specific DEGs between the two sides in females. Among the 159 DEGs, 138 were upregulated in the left while 21 were upregulated in the right. Note that only 8 DEGs were located on sex chromosomes. **D** GO analysis of the situs-biased genes in females

﻿The RNA-seq analysis of the four types of gonads revealed the genes differentially expressed between males and females, as well as between left and right in each sex. We identified 138 DEGs were female-biased and 163 DEGs were male-biased. Several ovarian developmental genes such as *FOXL2*, *CYP19A1*, *FSHR*, *HSD17B1* were presented among the female-biased genes. At the same time, a couple of testicular developmental genes such as *DMRT1*, *SOX9*, *AMH* were identified in the male-biased genes (Fig. 2A). This sex-biased gene expression pattern validated our sequencing results and indicated sexually dimorphic gene transcription pre-dating the phenotypic gonadal differentiation [31]. GO analysis of the sex-biased genes identified the biological processes related ovary and testis development such as “gonad development”, “development of primary sexual characteristics”, “reproductive structure development” and “sex differentiation” (Fig. 2B). These findings show that differentially expressed genes between sexes are indeed engaged in sexually dimorphic development of the gonads.

On the other hand, the comparison between left/right gonads within each sex yielded 159 DEGs (138 left-biased and 21 right-biased) in females (Fig. 2C) and only 49 DEGs (34 left-biased and 15 right-biased) in males (Additional file 8: Fig. S2). The GO analysis of the 159 situs-biased DEGs in females identified biological pathways related to gamete development such as “piRNA metabolic process”, “DNA methylation involved in gamete generation”, “cellular process involved in reproduction of multicellular organism” (Fig. 2D). It seems that the left female gonad was already intrinsically poised for further ovarian development and oocyte generation, although the two gonads were similar in appearance at this stage. In males, the overall transcriptome was comparable between the pair of gonads (Fig. 1A) and the 49 left/right DEGs were mainly involved in “positive regulation of protein phosphorylation” and “epithelial cell differentiation” pathways which were related to basic cell functions (Additional file 8: Fig. S2). Therefore, the further development of the gonad is well predicted by the comparisons of gene expression patterns and functional annotations between the pair of gonads in each sex.

### Elevated H3K27ac marks in the chromatin of left gonads in both sexes

The chromatin histone modification H3K27ac is associated with active transcription and it is found at both proximal and distal regions of transcription start site (TSS) of the expressed genes [32]. To gain insights into the epigenetic control of sex- and situs-specific gene expression and the subsequent gonad development, we sought to examine the active chromatin regions by scrutinizing H3K27ac occupancy on the genome from different gonads.

**Fig. 3:**
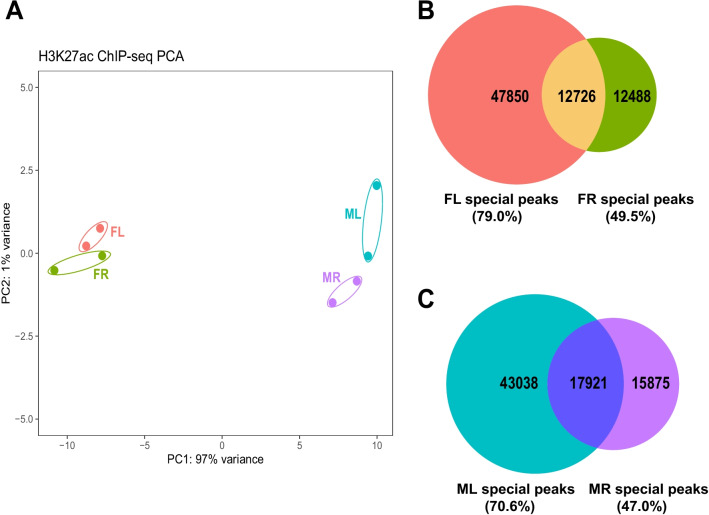
General analysis of H3K27ac ChIP-Seq data revealed that the active chromatin was divergent between left and right gonads in both sexes. **A** PCA of the ChIP-Seq datasets from the four types of gonads. FL: female left, FR: female right, ML: male left, MR: male right. **B** Venn diagrams indicating the number of H3K27ac peaks for each sample and the overlapped peaks between the left and right gonads in females. The percentage of left- and right-specific peaks among all the peaks in left and right female gonads were shown in brackets. **C** Venn diagrams indicating the number of H3K27ac peaks for each sample and the overlapped peaks between the left and right gonads in males. The percentage of left- and right-specific peaks among all the peaks in left and right male gonads were shown in brackets. Note the dramatic difference of H3K27ac peaks between left and right gonads were found in both females and males

ChIP-seq for H3K27ac was performed on two biological replicates and each replicate contained pooled gonadal somatic cells from multiple embryos. In general, the gonads from different sexes and sides can be nicely segregated by PCA analysis of the overall H3K27ac distributions (Fig. 3A). To our surprise, the left/right difference in males appears even more pronounced than that in females. It suggests that the two male gonads have divergent chromatin activities, albeit they are almost transcriptionally and morphologically indistinguishable. To corroborate the left/right discrepancy of H3K27ac profiles in different sexes, we further examined the shared and unique H3K27ac peaks among different gonads. Because each dataset from the same sample was highly reproducible since the correlation coefficient of the two replicates was higher than 96%, thus we combined the replicate files with unique comparisons for each replication for subsequent peak calling analysis (Additional file 3: Table S3, Additional file 9: Fig. S3).

In females, the previous transcriptional profiling showed that the left and right gonadal cells are already very distinct at this stage. Accordingly, the chromatin H3K27ac distributions were also different in the two female gonads. We identified considerable proportions of peaks were exclusively presented in female left and female right gonad, respectively (79% of left peaks (47,850/60,576) and 50% of right peaks (12,488/25,214)) (Fig. 3B). In other words, the two female gonads have diverged patterns of H3K27ac-modified active chromatin because the overlapping H3K27ac peaks between the left and right are very limited. In addition, the number of H3K27ac peaks in the female left is two times more than that in the female right, indicating that the overall chromatin activity is higher in the left than the right gonad. This is in consistent with the elevated gene expression and preferred development of the female left gonad. However, in males, we also found dramatic difference of H3K27ac distributions between the left and right. Similar to females, the male left gonad has much more H3K27ac peaks than the right one and there is limited overlap between them. We found that 70% of left peaks (43,038/60,959) are specific to the left gonad and 47% of right peaks (15,875/33,796) are specific to the right, respectively (Fig. 3C). Therefore, it seems that the left/right asymmetric genome occupancy of H3K27ac is presented in both females and males.

### Sex-specific H3K27ac chromatin modifications were associated with sex-biased gene expression and sex differentiation

**Fig. 4:**
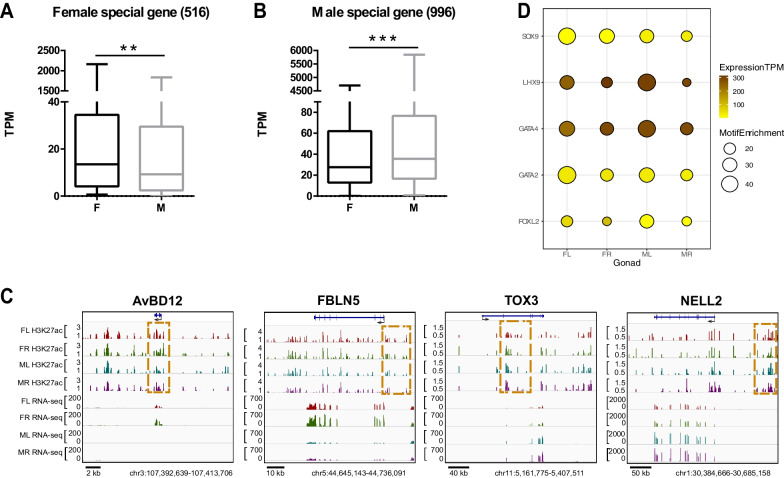
Positive correlation of sex-specific H3K27ac modifications on autosomal genes with the sex-biased gene expression. **A** The female-specific H3K27ac-associated genes are expressed higher in females. **B** The male-specific H3K27ac-associated genes are expressed higher in males. A total of 516 and 996 genes were identified to exclusively adjacent to the female- and male-specific H3K27ac peaks, respectively. The overall expression levels of the 516 genes are significantly higher in females than in males, while the overall expression of the 996 genes are significantly higher in males than in females. Kolmogorov Smirnov test was performed to compare the overall gene expression levels. **p < 0.01, ***p < 0.001. **C** Integrative Genomics Viewer (IGV) examples of H3K27ac binding and mRNA expression profiles of the selected autosomal genes showing sex-biased chromatin acetylation and gene expression. Note the *AvBD12* and *Fbln5* genes showed higher H3K27ac signal at the proximal regulatory regions and elevated mRNA expression levels in female gonads, whereas *Tox3* and *Nell2* genes showed higher H3K27ac signal at the neighboring regulatory chromatin regions and elevated mRNA expression levels in male gonads. The H3K27ac track represents the peak signal after subtracting the non-specific input. **D** The binding motif enrichment analysis of H3K27ac marked chromatin regions from different gonads. Note the binding motifs of the male-specific (Sox9) and female-specific (Foxl2) transcription factors were enriched in the H3K27ac marked chromatin regions in both sexes, although *Sox9* and *Foxl2* already show male- and female-biased expression, respectively

To validate the functional relevance of the H3K27ac marked chromatin activation, we examined whether the differentially distributed H3K27ac peaks were also associated with differential expression of genes associated with gonad differentiation. We annotated the genes proximal to the H3K27ac peaks using a window of ± 15 kb from the transcription start site (TSS) and gene body. After duplication removal, we obtained the peak-related genes in different gonads (8087 in FL, 5475 in FR, 9391 in ML and 6887 in MR, Additional file 4: Table S4). To determine whether the sex-specific H3K27ac chromatin modifications could contribute to sex-biased gene expression, we compared the expression levels of peak-related genes that are unique to each sex. We identified 516 and 996 genes that are exclusively adjacent to the female- and male-specific H3K27ac peaks, respectively (Additional file 5: Table S5). The overall expression levels of the 516 genes associated with the female-specific peaks are significantly higher in females when compared to males (Fig. 4A). Similarly, the 996 genes adjacent to male-specific peaks showed significant up-regulation in males than that in females (Fig. 4B). Thus, the sex-specific H3K27ac chromatin modifications were strongly associated with the female- or male-specific gene up-regulation in each sex. The RT-qPCR also confirmed the differential expression of the sex-biased genes (Additional file 10: Fig. S4).

Next, we carefully examined the positive correlation between H3K27ac chromatin modification and sex-related gene expression through the Integrative Genomics Viewer (IGV) visualization analysis (Fig. 4C). Among the female-specific peak-related genes, the *AvBD12* (Avian β-Defensin 12) gene is highly expressed in developing ovary, such as the granulosa and theca cells in the follicle and the perivitelline layer of the oocytes [33, 34]. We found the H3K27ac signal was much more enriched in the promoter and intron regions of *AvBD12* locus in females than that in males, and correspondingly the *AvBD12* gene expression level is much higher in female gonads than in males. We also identified other sex-specific H3K27ac-associated proximal genes (*Fbln5*, *Nell2*, *Tox3*) that could be involved in either ovary or testis development, as shown by their sex-biased mRNA expression (Additional file 11: Fig. S5). The female-biased *Fbln5* was involved in ovarian epithelial cell proliferation and transformation [35, 36]. The male-biased *Nell2* is specifically expressed in the developing testis and produced a secreted luminal protein to stimulate epididymal differentiation and sperm maturation [37]. Another male-biased gene *Tox3* is a novel Sertoli cell marker co-expressed with *Sox9* in developing chicken testis cords [38]. Therefore, the sex-specific chromatin activation can contribute to the sex-biased gene expression and promote the development of either ovary or testis.

Meanwhile, the transcription factor binding motif analysis of the H3K27ac peaks in different gonads could help us to identify the potential transcription factors that can engage in the sex-specific chromatin activation and sex gene regulation. Figure 4D shows the most enriched binding sites for transcription factors related to sex differentiation. Interestingly, the binding sites of male and female-specific transcription factors are presented in both sexes at comparable frequencies. For example, the binding motifs of *Sox9* (testis-specific gene) and *Foxl2* (ovary-specific gene) were enriched in the H3K27ac peaks from both sexes, but the expression of the two genes was already skewed towards each corresponding sex. Therefore, it seems that the active chromatin regions of sexually bi-potential gonads were accessible to the core sex-determining transcription factors of both sexes and thus could potentiate sex differentiation toward either one of the two directions. In other words, in the undifferentiated bi-potential gonads, the sex-specific chromatin activation could contribute to the sex-biased expression of proximal genes, but the active chromatin regions could be potentially targeted by sex-determining transcription factors of either sex.

### The H3K27ac occupancy on sex chromosomes does support the dosage effect of Z but regional compensation could also be identified

**Fig. 5:**
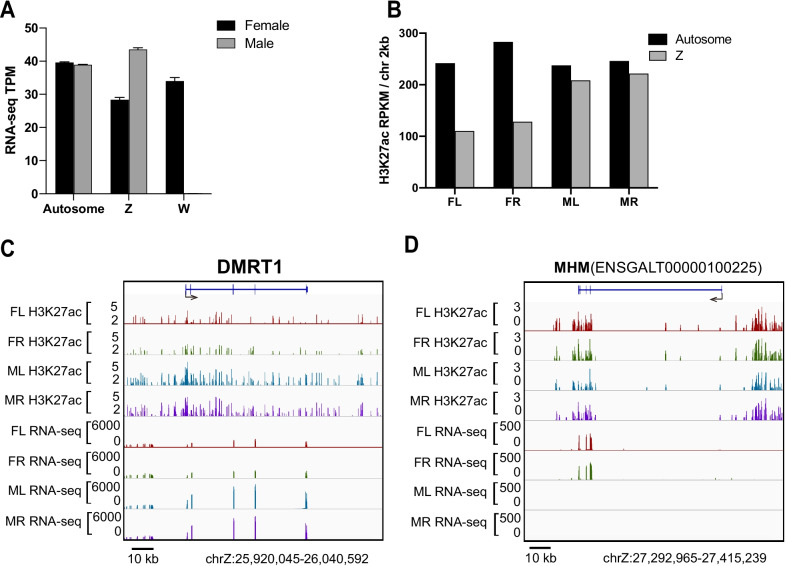
The overall H3K27ac distribution on Z chromosome is higher in ZZ males than in ZW females. **A** The average expression levels of all genes from autosome, Z and W chromosomes in different sexes. The autosome gene expression was comparable between two sexes. The general expression level of Z genes was almost two times higher in ZZ males than in ZW females. The W genes were expressed only in ZW females. **B** The average H3K27ac modification level across the whole Z chromosome genes is almost two times higher in males than in females. H3K27ac ChIP BAM files were used to calculate the reads coverage for autosome and sex chromosomes. The coverage calculation is done for consecutive bins of 2 kilobases. **C** The IGV shows that both the H3K27ac occupancy near *Dmrt1* locus and the expression level of *Dmrt1* mRNA is higher in males than females. Note the widespread deposition of H3K27ac around *Dmrt1* locus in both sexes but much higher intensity was observed in males. **D** The IGV indicates that regulatory region of MHM locus is dosage compensated as shown by the higher H3K27ac signal in ZW females than in ZZ males. The non-coding RNA is only transcribed from the single Z chromosome in females but not expressed in males

In birds, the dosage effect, but not the dosage compensation (chromosome inactivation), of the sex chromosomes was proposed to play a major role in the sex differentiation of the bi-potential gonads [39]. Accordingly, we found the Z chromosome genes are in general expressed at a higher level in gonads of males (ZZ) than females (ZW) (Fig. 5A), prompting us to examine the chromatin activation levels of the Z chromosome between different sexes. We quantified the global occupancy level of H3K27ac in both sex chromosomes and autosomes from both sexes. As shown in Fig. 5B, the average H3K27ac signal across the whole Z chromosome in males is almost twofold of that in females. It seems that both copies of the Z chromosomes in the males are active and therefore double H3K27ac intensity of Z in males than in females can be detected by ChIP-seq. Hence, the overall male/female ratio of chicken Z chromosome activity does support the “gene dosage effect” hypothesis of which gene copy numbers predominantly determine the gene expression.

We also examined the H3K27ac distribution on several important regions on the Z chromosome to see if there are specific dosage-compensated regions. In theory, the ZZ males would exhibit double dose intensity of H3K27ac peaks compared to the ZW females at specific locations assuming each Z chromosome is equally modified by H3K27ac across the whole chromosome. The *Dmrt1* (doublesex and mab-3-related transcription factor 1) is the key male determining gene located on Z chromosome [39, 40]. As expected, around the *Dmrt1* locus the male gonads showed much higher H3K27ac signal than females and so does the mRNA expression level of the *Dmrt1* gene (Fig. 5C). On the other hand, the Z-derived MHM (male hypermethylated) locus is methylated and thus silent in male gonads, but transcribes a female-specific non-coding RNA that is involved in gonadal sex differentiation [41]. Accordingly, we observed a higher H3K27ac signal in ZW females than ZZ males around the regulatory region of MHM locus (Fig. 5D). Thus, the local dosage compensation in specific loci, such as the MHM locus, was also evidenced by the H3K27ac marked chromatin activity [42].

### Situs-specific H3K27ac occupancy was associated with the left/right asymmetric gene expression in females but not males

**Fig. 6:**
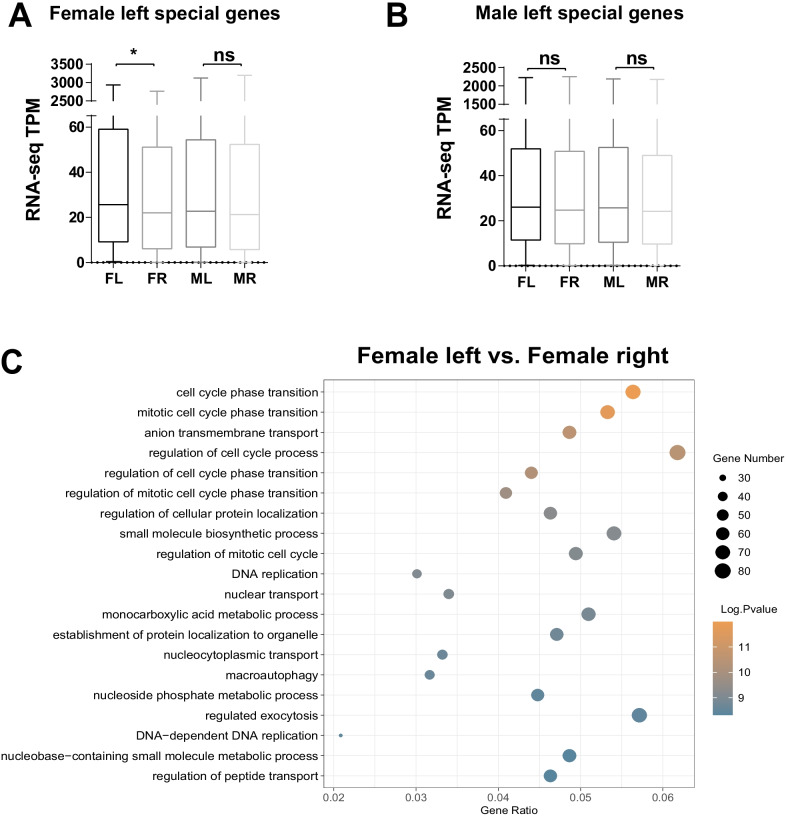
Left-specific H3K27ac occupancy coincides with higher gene expression in left gonad in females but not males. **A** The overall expression levels of female left-specific H3K27ac-associated genes (*n* = 1555) among different gonads. Note the average mRNA levels of female left-specific H3K27ac-associated genes were significantly higher in the female left than the female right. But in males, these genes were equally expressed. Kolmogorov–Smirnov test was performed to compare the overall gene expression levels. *p < 0.05, ns: not significant. **B** The expression levels of male left-specific H3K27ac-associated genes (*n* = 1552) among different gonads. The mRNA levels of male left-specific H3K27ac-associated genes were not different between left and right gonads in either sex. **C** The GO analysis of the female left-specific H3K27ac-associated genes indicates that cell cycle and ovarian development-related pathways were enriched

Next, we want to know whether the situs-specific H3K27ac distribution also leads to the differential gene expression between left and right gonads in each sex. We focused on the left-biased H3K27ac marks and the associated genes since much higher H3K27ac modification levels were detected at the left gonad than the right one in both sexes. We obtained 1555 genes which were accompanied with left-specific H3K27ac peaks in females and the expression of these genes was significantly higher in the left gonad compare to the right gonad in females. But in males these female left-specific H3K27ac-associated genes were expressed equally between left and right gonads (Fig. 6A, Additional file 5: Table S6). We also identified 1552 genes adjacent to the left-specific H3K27ac peaks in males through the same approach, but the mRNA levels of these genes were similar between left and right gonads in both sexes (Fig. 6B, Additional file 6: Table S6). Therefore, a positive correlation was identified between the situs-specific H3K27ac chromatin modifications and the neighboring gene expression in female gonads, but in males the situs-specific H3K27ac occupancy does not affect the overall expression level of the associated genes. Nevertheless, the Pitx2 gene, the master regulator of left/right axis development in vertebrate embryos [43, 44], showed much higher H3K27ac deposition and mRNA expression in the left compared to the right in both sexes (Additional file 12: Fig. S6). Hence, diverse molecular mechanisms could engage in the left/right asymmetric gonadal development regulation at different levels.

The GO analysis of the 1555 female left-specific H3K27ac-associated genes revealed that several cell cycle-related pathways were most enriched, such as the "cell cycle phase transition", "mitotic cell cycle phase transition" and "DNA replication" (Fig. 6C). We speculated that in the female, the left-specific H3K27ac chromatin modifications support the chromatin activation and the elevated expression of cell cycle genes which lead to further cell proliferation and gonad development. In contrast, the diminished H3K27ac distribution in the right gonad could lead to the downregulation of the developmental genes and cause the atrophy of the right gonad in females. While in males, other mechanisms may override the left/right H3K27ac distribution discrepancy to control the expression of genes necessary for development equally at both sides, so that the male right gonad can grow normally as the left one.

### Trichostatin A-induced histone acetylation retention in the female right gonad could rescue the expression of ovarian genes and stimulate gonadal cell proliferation

**Fig. 7:**
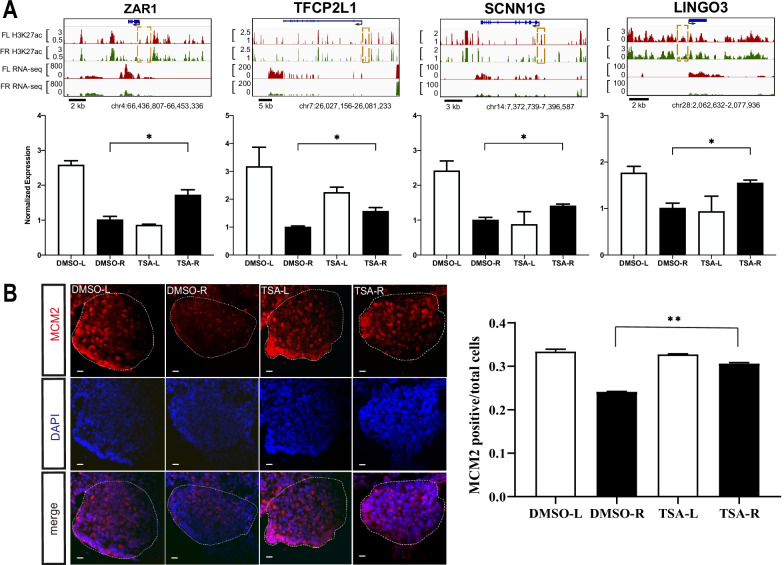
The HDAC inhibitor trichostatin A treatment in the female gonads increased the expression of ovarian genes and gonadal cell proliferation. **A** Representative IGV browser view of *Zar1*, *Lingo3*, *Scnn1g* and *Tfcp2l1*, which are female left-specific H3K27ac-associated genes (top). Note the enriched H3K27ac depositions around the regulatory regions of the selected genes and the higher mRNA expression in the female left comparing to the female right. Quantitative PCR verification of the gene expression levels after TSA treatment (bottom). Data were mean ± SEM, *n* = 4, * *p* < 0.05 by Student’s *t* test. **B** Representative images of immunofluorescence staining of the cell proliferation marker MCM2 in different female gonads treated with TSA or DMSO. Note the TSA-treated female right gonad showed increased cell proliferation compared to the DMSO treated female right gonad. The proportions of MCM2^+^ cells were quantified from each group of gonads (*n* ≥ 5). Data were mean ± SEM, ** *p* < 0.01 by Student’s t test. Scale bar: 20 μm

Trichostatin A (TSA) is a potent and reversible inhibitor of histone deacetylase (HDAC), therefore acting as an epigenetic modifier by preventing the removal of acetyl groups from lysine residues on histone tails. TSA can be used to selectively promote gene transcription by maintaining chromatin histone H3K27 acetylation [45]. At E4.5, we administered the TSA or DMSO into the right mesonephric kidney where the early gonad originated and examined the expression levels of the situs-biased genes 24 h later (Additional file 13: Fig. S7). Since the right gonad naturally stopped growing and regressed hereafter, we therefore determined the effects of TSA-induced H3K27ac retention and chromatin activation on rescuing the ovarian gene expression and gonadal development. We first confirmed that the H3K27ac deposition is indeed elevated upon TSA treatment in the female right gonad (Additional file 14: Fig. S8). By real-time PCR analysis of several situs-biased genes, we found that TSA treatment could stimulate the transcription of the ovarian genes in the regressing gonad (Fig. 7A). For example, zygote arrest 1 (*ZAR1*) is an evolutionarily conserved gene expressed in vertebrate ovaries and play essential roles during ovarian development [46]. In chicken, the *Zar1* is involved in the gonadal development as well as the maturation of ovary and oocyte [47, 48]. Based on the current RNA-seq and ChIP-seq in females, we identified that *Zar1* is expressed significantly higher in the developing gonad than the regressing one and the left-specific H3K27ac occupied chromatin at the regulatory region could be responsible for the asymmetrical transcription (Fig. 7A). The TSA administration in the right gonad stimulated the chicken *Zar1* transcription to even exceed the expression level of the left gonad. Similarly, the expression level of other reproductive and developmental genes, such as *Lingo3* [49], *Scnn1g* [50], *Tfcp2l1* [51], were all elevated to the comparable levels of the developing left gonad (Fig. 7A). Therefore, induced chromatin acetylation seems to be able to rescue some of the ovarian gene expression in the regressing female right gonad.

To ascertain whether the induced ovarian gene expression could be translated into functional growth of the right gonad, we also examined the cell proliferation by immunostaining of cell cycle markers in TSA and DMSO treated embryos. The expression and localization of cell proliferation marker MCM2 (Mini-Chromosome Maintenance Protein 2) in the gonads was investigated (Fig. 7B). The abovementioned GO analysis of the female situs-specific H3K27ac-associated genes indicated that cell cycle-related pathways were most enriched in the left growing gonad compare to the right regressing one. Accordingly, the pair of the control gonads (DMSO treated group) in females displayed the left-biased proliferative MCM2 distribution pattern as well as the left-enriched ovarian genes expression. However, in TSA-treated group, the proportion of MCM2^+^ cells in the right gonad increased to levels comparable to the left (Fig. 7B). This demonstrates that impaired cell proliferation in the right gonad could be rescued by forcefully chromatin acetylation. Hence, the lack of chromatin activation could be responsible for the cellular growth defects in the female right gonad at the early developmental stage.

## Discussion

Gonadal sex differentiation is induced by the genetic or epigenetic switch that activates one pathway and represses the other, making both sex determination processes mutually exclusive. To date, researchers generally believed that the dosage effects of the Z chromosome determined the genetic sex of the chicken gonad [39]. The ZZ males have two copies of Z-linked *Dmrt1* which is expressed higher and then induced more Sox9 expression than ZW females and subsequently initiate the cascade of the testicular program [11, 39]. We noticed the distribution of H3K27ac was substantially enriched around *Dmrt1* locus in both males and females. Hence the completely activated chromatin status around *Dmrt1* locus in both sexes could ensure the precise control of *Dmrt1* expression levels solely based on the amount of DNA template. In other words, the chromatin histone modifications should not interfere with the chromatin dosage effects at this important and sensitive locus. Nevertheless, the H3K27ac modifications around MHM region suggest that some specific regions in Z chromosome could also be dosage compensated. With the current techniques we cannot determine the H3K27ac occupancy level in each single Z chromosome of the ZZ gonad and the possibility of biased chromatin activity between the two Z chromosomes still exist. But it is highly unlikely that one Z has much higher H3K27ac than the other one in males.

In addition to the sex-biased H3K27ac distribution on sex chromosomes, our ChIP-Seq data also revealed many autosome-located sex-determining genes could be regulated by chromatin activities. We showed that the male- and female-specific acetylated chromatin regions were associated with the elevated expression of adjacent genes in a sex-specific manner. Namely, the male-specific H3K27ac peaks were enriched around testis-promoting genes and female-specific H3K27ac peaks were enriched around ovary-promoting genes. For instance, the expression levels of genes proximal to female-specific H3K27ac peaks were significantly higher in females than males. Many of these genes, such as *AvBD12* [34] and *Fbln5* [36], were necessary for ovarian development. This is also the case in males, as shown by the distribution of male-specific H3K27ac peaks in the vicinity of testicular developmental genes. Therefore, the sex-biased chromatin activities marked by H3K27ac could facilitate the expression of sex differentiation genes in each sex and initiate the bi-potent gonads to develop into either ovaries or testes eventually.

Besides the male/female asymmetry, the chicken gonad development also shows another aspect of asymmetry along the left/right axis which is unique among vertebrates. In females, the left gonad will develop into a functional ovary and the right gonad will regress gradually [5]. At the undifferentiated stage, the appearance of the pair of female gonads looked similar macroscopically [52], but they already exhibit a dramatic difference in gene expression as shown by our and other transcriptome analyses [29, 38]. The female left gonad expresses much more developing genes compared to the right one [31] and the differential gene transcription largely corresponded to the expected direction based on H3K27ac status between the left and right. Thus, in females, the left-specific chromatin activation coincides well with ovarian gene up-regulation and we can postulate that the H3K27ac-modified active chromatin regions could play a major role in left/right asymmetric development. Further, to test the hypothesis that the left-specific (or right-lost) histone acetylation of the chromatin caused the asymmetric gene expression and unilateral gonadal development, we purposely manipulated the chromatin acetylation levels and examined the ovarian gene expression as well as the gonadal cell proliferation in the right gonads of females. Trichostatin A (TSA) induced histone acetylation could alter the chromatin and transcriptional activity in a variety of tissue and cell systems [45]. In TSA-treated female gonads, we observed bilateral comparable expression of several ovarian genes which were originally left-biased in DMSO-treated gonads, although the relative expression levels varied from being comparable to even higher on the right compared with the left side. Thus, forcefully elevating the chromatin of right gonadal cells could rescue the expression of ovarian genes which were weakly transcribed originally. Further, the development of the right gonad was also partially recovered as shown by the increased cell proliferation after TSA treatment.

Next, we analyzed the chromatin acetylation patterns in left and right gonads in males. To our surprise, we also found dramatic differences in H3K27ac distribution patterns between the male left and male right, although the morphological appearance and the overall transcriptome of the two gonads are very similar [52]. Comparable to the females, the male left gonad exhibits much stronger H3K27ac modifications than that in the male right. But the overall expression levels of the genes proximal to the left-biased H2K27ac were equal between left and right in males. In other words, the positive correlation or trend between the left-biased H3K27ac depositions and the overall higher expression of proximal genes exists in females but not in males. We propose that other mechanisms can override the chromatin activity switch to directly induce the testicular gene expression in both sides of the male gonads. Currently, we do not know if the mammalian gonads will also show asymmetrical chromatin activity between left and right in either sex.

### Perspectives and significance

Current research increases our understanding of the complex regulatory network underlying the sex determination and situs differentiation of gonads in birds. The undifferentiated left and right gonads are macroscopically indistinguishable in both female and male. In females, the specified H3K27ac marked chromatin state of gonadal progenitor cells largely coincides with the sex- and situs-biased gene expression patterns in each side of gonads and could contribute to the asymmetrical development of the gonads. In males, the two gonads exhibit similar gene expression patterns and development potential but extensive left/right discrepancy in chromatin activity, and future research is needed to identify the underlying mechanisms overruling this discordancy. Our study also provides a powerful genetic and epigenetic resource for identifying the regulatory elements that could be harnessed for sex modulation in poultry industry as well as the understanding of sex-related disorders.

## Supplementary Information

**Additional file 1: Table S1.** List of primers used for real-time RT-qPCR assay.

**Additional file 2: Table S2.** The information and mapping summary of RNA-Seq & ChIP-Seq sequencing.

**Additional file 3: Table S3.** The detailed DEGs and H3K27ac peaks from RNA-Seq & ChIP-Seq sequencing.

**Additional file 4: Table S4.** The detailed list of H3K27ac peak adjacent genes after the removal of duplicates.

**Additional file 5: Table S5.** The peak and expression data of the sex-specific H3K27ac chromatin modifications and the associated genes.

**Additional file 6: Table S6.** The peak and expression data of the situs-specific H3K27ac chromatin modifications and the associated genes.

**Additional file 7: Figure S1.** Correlation test between the biological replicates of RNA-Seq data. (A) Female left. (B) Female right. (C) Male left. (D) Male right.

**Additional file 8: Figure S2.** GO analysis of the 49 left–right DEGs in males.

**Additional file 9: Figure S3.** Correlation test of ChIP-Seq data. (A-D) Correlation test between the biological replicates within the same gonad. (E–H) Correlation test between the combined data and inputs from the same gonad. Note that the correlation between biological repeats is high and the correlation between IP and input is low.

**Additional file 10: Figure S4.** The RT-qPCR verification of the differential expression of sex-biased genes. Data was mean ± SEM, n ≥ 4, * p < 0.05, ** p < 0.01, *** p < 0.001 by Student t test.

**Additional file 11: Figure S5.** (A) The expression pattern of sex-biased genes during gonad development. The RNAseq data were from male and female gonads of E4.5, E6, E10, E12, E14. (B) IGV example of AvBD12 locus showing the H3K27ac peaks including the input tracks accompany with the RNA-seq reads.

**Additional file 12: Figure S6.** The IGV presentation of Pitx2 gene showed that both the H3K27ac deposition and the mRNA expression are higher in the left than in the right in both males and females.

**Additional file 13: Figure S7.** The flowchart of TSA injection experiments. (A) Schematic overview of TSA injection and experimental design. (B) Photo of TSA injection into the female right mesonephric region.

**Additional file 14: Figure S8.** (A) Representative images of immunofluorescence staining of the H3K27ac distribution in the female left gonads treated with TSA or DMSO. Note the TSA treated female right gonad showed increased and punctate H3K27ac signal compared to the DMSO. Scale bar: 20 μm. (B) The quantifications of the H3K27ac positive nuclei in the gonads of different treatments. Data was mean ± SEM, n = 4, * p < 0.05 by Student t test.

## Data Availability

All data are available from the corresponding author upon request.
