# Supplementary material for: H3K27ac chromatin acetylation and gene expression analysis reveal sex- and situs-related differences in developing chicken gonads
Source: Biol Sex Differ. 2022 Feb 8;13:6. doi: 10.1186/s13293-022-00415-5 (PMC8822763; doi:10.1186/s13293-022-00415-5)
Supplement: Supplementary file 1 — Additional file 1: Table S1. List of primers used for real-time RT-qPCR assay. [file 13293_2022_415_MOESM1_ESM.docx]

**Additional file 1: Table S1.** List of primers used for this study

| Gene name | Forward | Reverse |
| --- | --- | --- |
| AvBD12 | GATGGGGATCCGGCCCAGACAGCTGTAAC | TCCATGGTACCTCAGGTCTTGGTGGGAG |
| HINTW | ACCGCGCACATCTCTGTATT | CGTGAAATCCATTCGGTGGC |
| ACAT2 | TGCACTGGAACGAACAGGTG | AAGCCTCTGTCATCGTAGGGA |
| RRAD | CAAGCTGCGCTACCTAGACA | GAGGAGATGACGGAGTCGGA |
| ZAR1 | CACTGCAAGGACTGCAAC | CTCTGGCAGGTGATGTCCTC |
| LINGO3 | AGGAGAGCACCTTCCACTCT | GAAGTCACGCAGAGCGTTTC |
| SCNN1G | CTGTTCTTCAGGCATGTCCCA | TGGACGAGTCCTTGTGGAAGA |
| TFCP2L1 | ACTGCCTCCGTTTCAGTACG | TGGTTGAGGTAGGTCAGGGT |
| PCK1 | GCAGGGGTTATGATGAGAAGT | ACGGATCACAGTTTTGAAGAC |
| PITX2 | TGCTCCTCCTCACCTTCCTC | ATCCTCGCTCTTGCCCTG |
| GAPDH | CCTCTCTGGCAAAGTCCAAG | CATCTGCCCATTTGATGTTG |
| CHD1 | TGCAGAAGCAATATTACAAGT | AATTCATTATCATCTGGTGG |
