# Supplementary material for: H3K27ac chromatin acetylation and gene expression analysis reveal sex- and situs-related differences in developing chicken gonads
Source: Biol Sex Differ. 2022 Feb 8;13:6. doi: 10.1186/s13293-022-00415-5 (PMC8822763; doi:10.1186/s13293-022-00415-5)
Supplement: Supplementary file 2 — Additional file 2: Table S2. The information and mapping summary of RNA-Seq & ChIP-Seq sequencing. [file 13293_2022_415_MOESM2_ESM.docx]

**Additional file 2: Table S2.** The information and mapping summary of RNA-Seq & ChIP-Seq sequencing data

| Data type | Factor | ID | Total reads | Total mapped rate | Unique mapped rate |
| --- | --- | --- | --- | --- | --- |
| RNA-Seq | None | Female Left-1 | 23,350,490 | 94.59% | 88.76% |
| RNA-Seq | None | Female Left-2 | 21,569,286 | 94.60% | 88.41% |
| RNA-Seq | None | Female Right-1 | 21,606,511 | 95.02% | 89.30% |
| RNA-Seq | None | Female Right-2 | 23,086,643 | 94.65% | 88.80% |
| RNA-Seq | None | Male Left-1 | 23,040,018 | 94.87% | 89.70% |
| RNA-Seq | None | Male Left-2 | 21,468,592 | 95.00% | 89.44% |
| RNA-Seq | None | Male Right-1 | 21,974,182 | 94.93% | 89.66% |
| RNA-Seq | None | Male Right-2 | 21,880,113 | 94.91% | 89.61% |
| ChIP-Seq | H3K27ac | Female Left-1 | 34,162,904 | 93.83% | 89.00% |
| ChIP-Seq | H3K27ac | Female Left-2 | 39,949,072 | 97.04% | 92.70% |
| ChIP-Seq | H3K27ac | Female Right-1 | 54,628,907 | 92.06% | 86.74% |
| ChIP-Seq | H3K27ac | Female Right-2 | 50,644,942 | 94.45% | 89.94% |
| ChIP-Seq | H3K27ac | Male Left-1 | 41,892,038 | 84.65% | 80.29% |
| ChIP-Seq | H3K27ac | Male Left-2 | 56,166,170 | 96.44% | 92.42% |
| ChIP-Seq | H3K27ac | Male Right-1 | 31,641,203 | 92.15% | 87.68% |
| ChIP-Seq | H3K27ac | Male Right-2 | 47,882,278 | 97.43% | 92.58% |
| ChIP-Seq | None | Input | 35,303,804 | 85.60% | 77.52% |
