# Supplementary figures and images for: H3K27ac chromatin acetylation and gene expression analysis reveal sex- and situs-related differences in developing chicken gonads
Source: Biol Sex Differ. 2022 Feb 8;13:6. doi: 10.1186/s13293-022-00415-5 (PMC8822763; doi:10.1186/s13293-022-00415-5)

**A**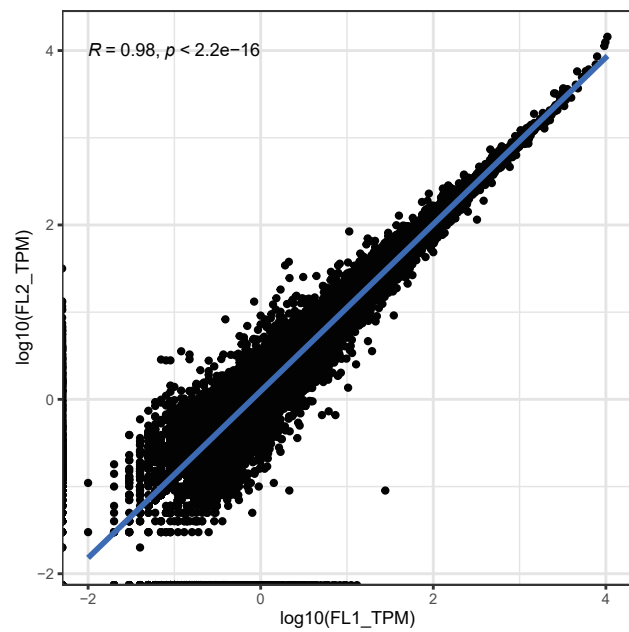**B**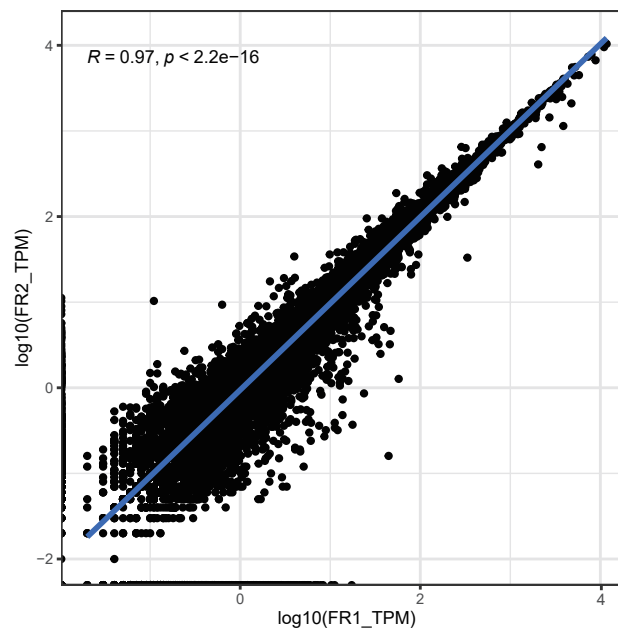**C**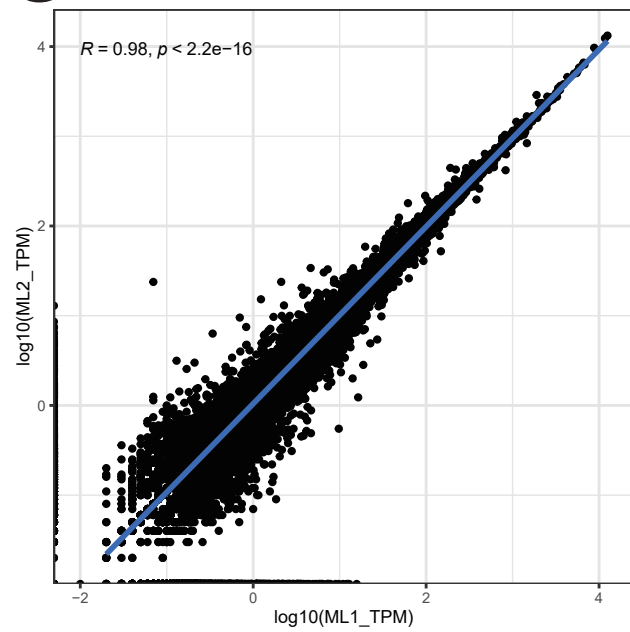**D**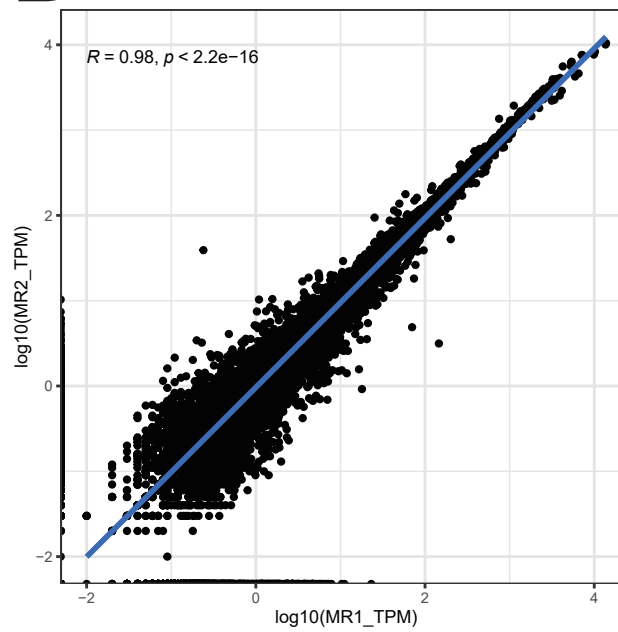

Supplement: Supplementary file 7 — Additional file 7: Figure S1. Correlation test between the biological replicates of RNA-Seq data. (A) Female left. (B) Female right. (C) Male left. (D) Male right. [file 13293_2022_415_MOESM7_ESM.pdf]

# Male Left vs Male Right

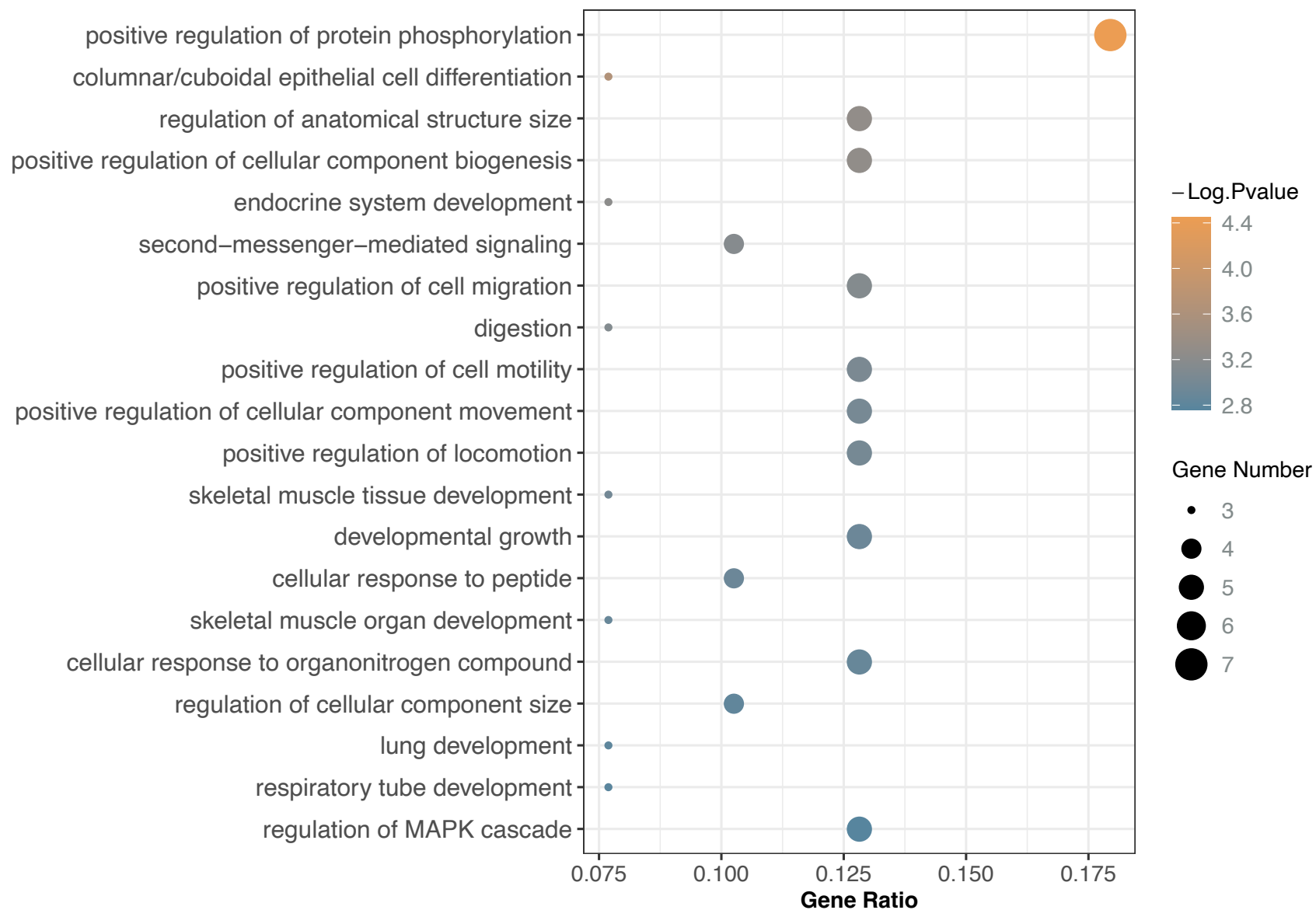

Supplement: Supplementary file 8 — Additional file 8: Figure S2. GO analysis of the 49 left–right DEGs in males. [file 13293_2022_415_MOESM8_ESM.pdf]

**A**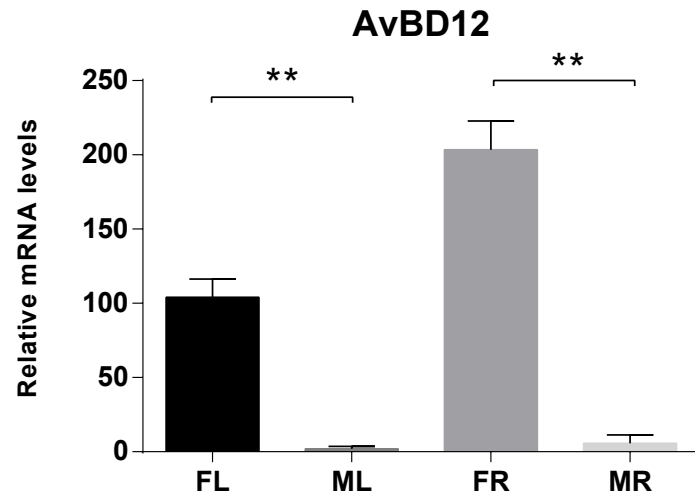**B**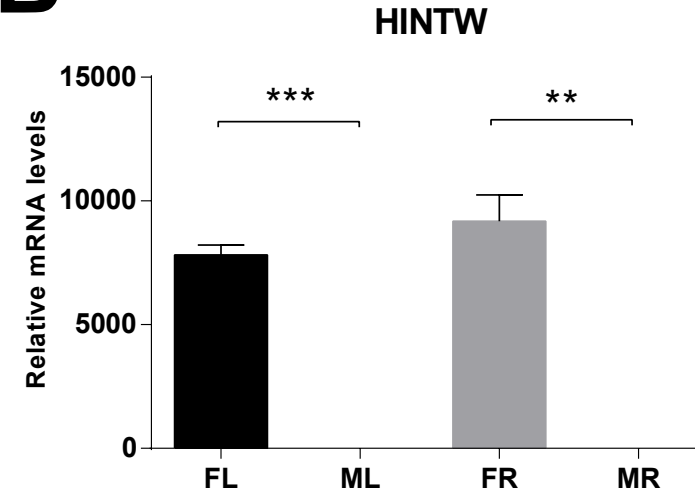**C**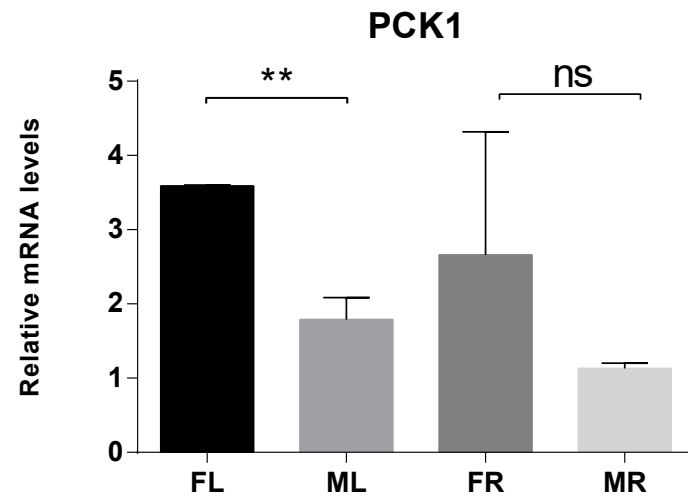**D**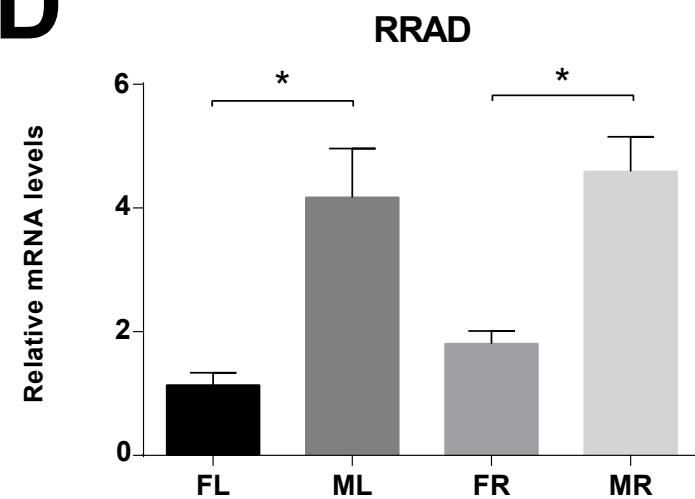

Supplement: Supplementary file 10 — Additional file 10: Figure S4. The RT-qPCR verification of the differential expression of sex-biased genes. Data was mean ± SEM, n ≥ 4, * p < 0.05, ** p < 0.01, *** p < 0.001 by Student t test. [file 13293_2022_415_MOESM10_ESM.pdf]

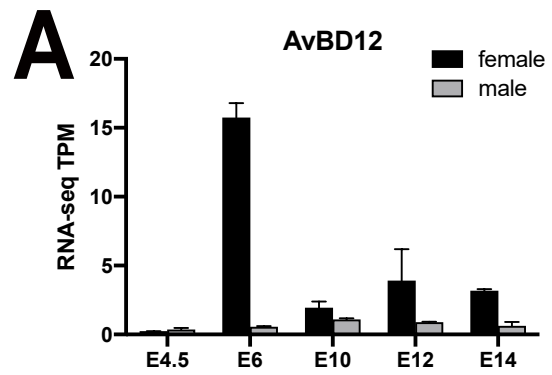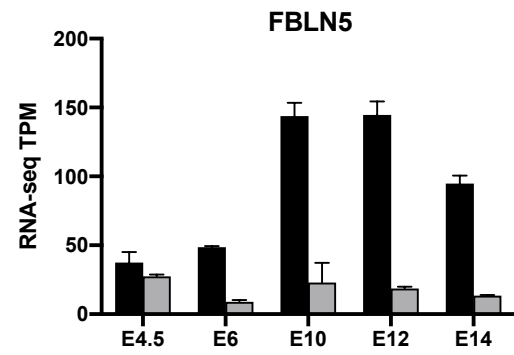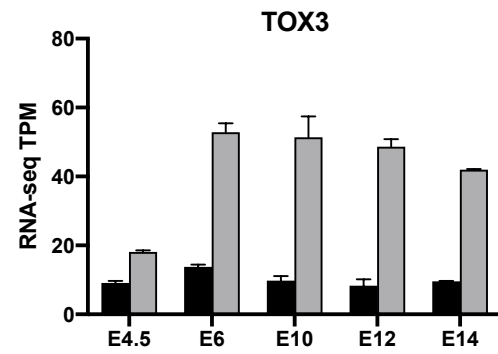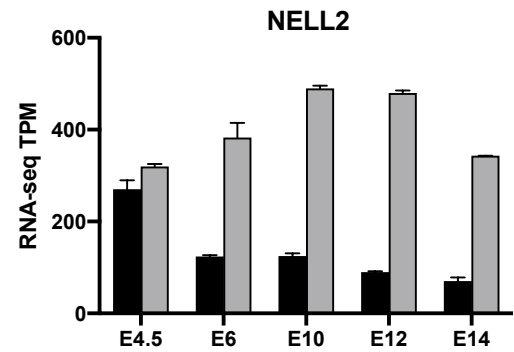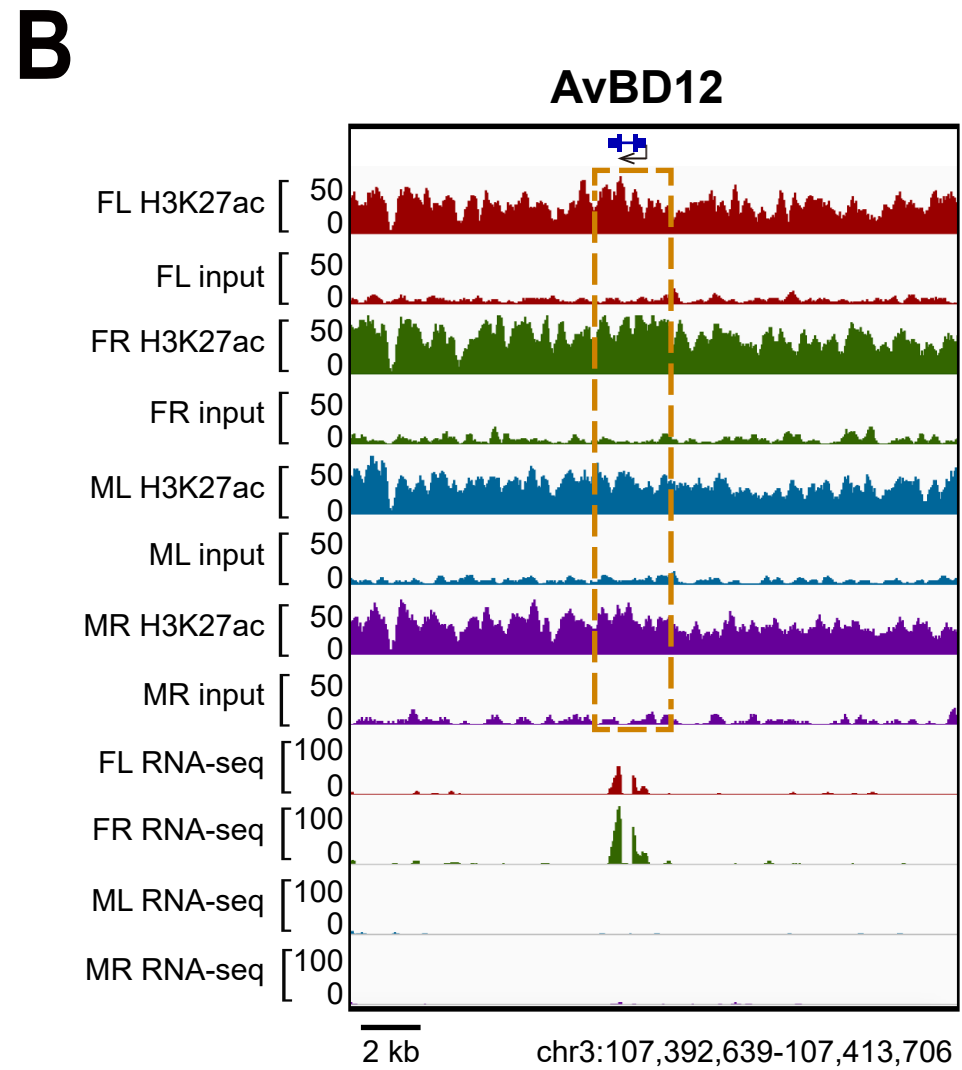

Supplement: Supplementary file 11 — Additional file 11: Figure S5. (A) The expression pattern of sex-biased genes during gonad development. The RNAseq data were from male and female gonads of E4.5, E6, E10, E12, E14. (B) IGV example of AvBD12 locus showing the H3K27ac peaks including the input tracks accompany with the RNA-seq reads. [file 13293_2022_415_MOESM11_ESM.pdf]

**A**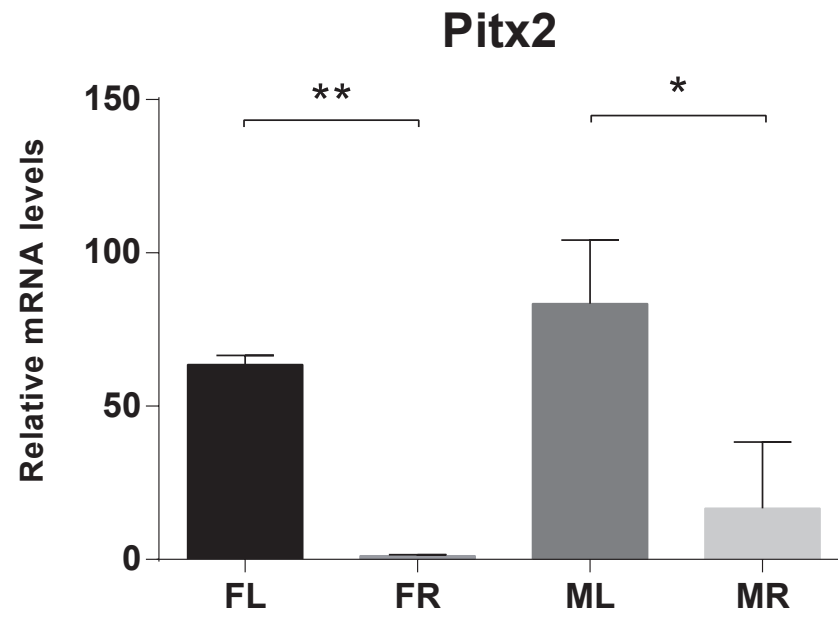**B**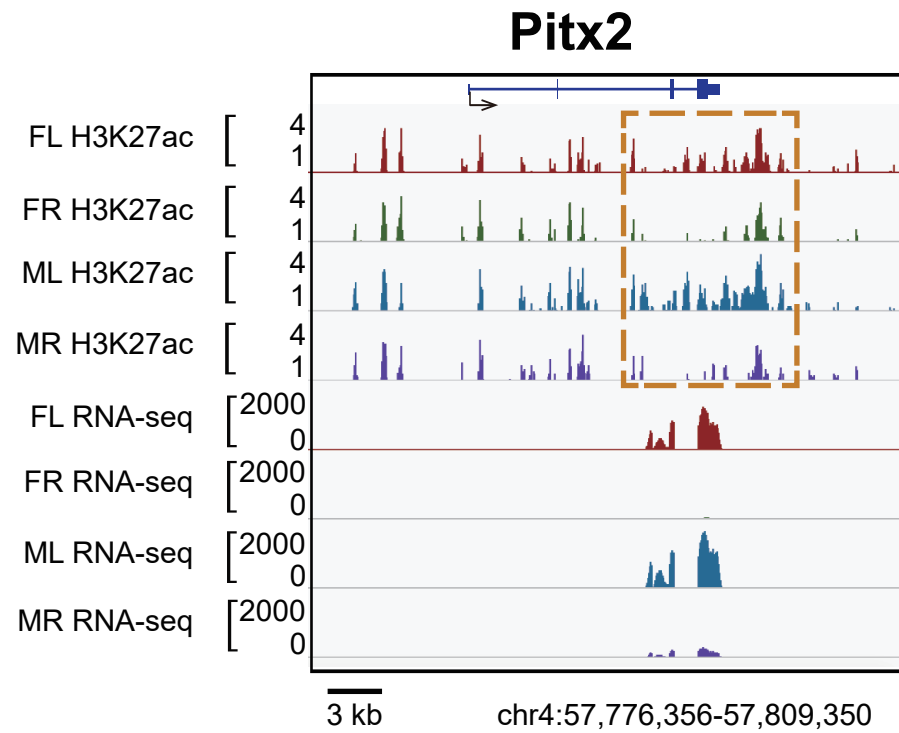

Supplement: Supplementary file 12 — Additional file 12: Figure S6. The IGV presentation of Pitx2 gene showed that both the H3K27ac deposition and the mRNA expression are higher in the left than in the right in both males and females. [file 13293_2022_415_MOESM12_ESM.pdf]

**A**

TSA or DMSO

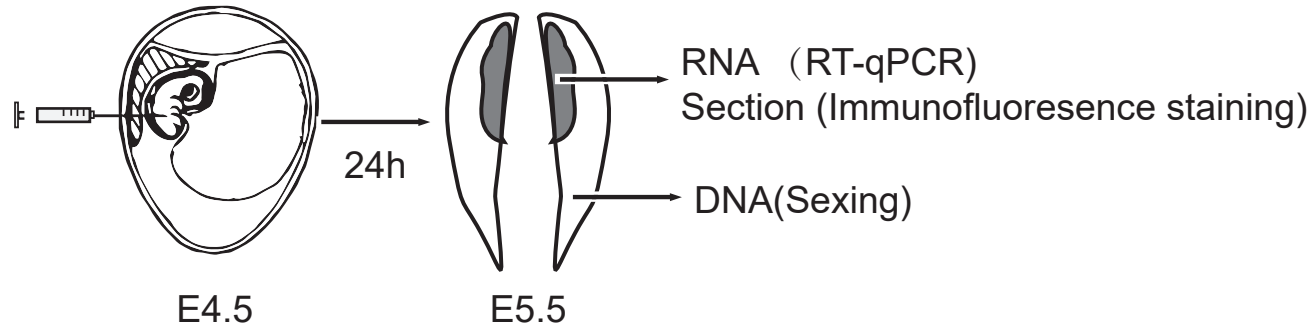**B**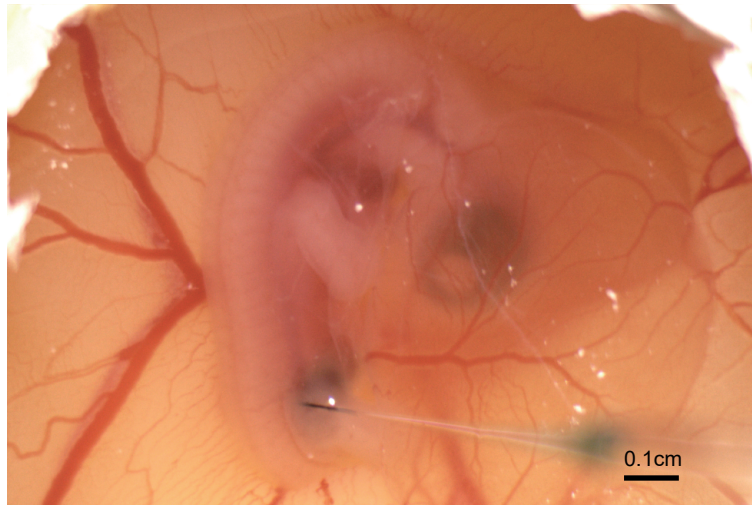

Supplement: Supplementary file 13 — Additional file 13: Figure S7. The flowchart of TSA injection experiments. (A) Schematic overview of TSA injection and experimental design. (B) Photo of TSA injection into the female right mesonephric region. [file 13293_2022_415_MOESM13_ESM.pdf]

**A**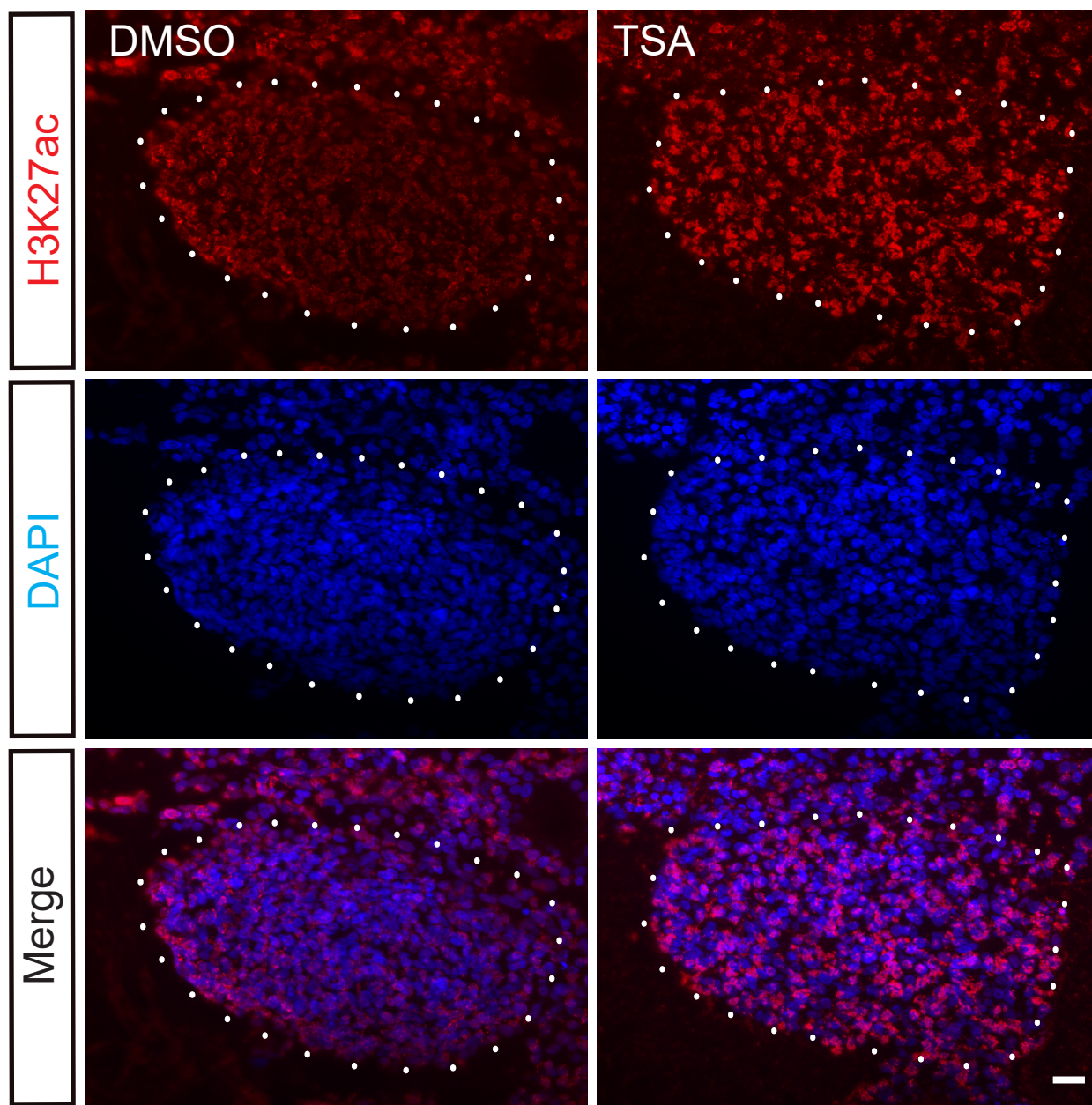**B**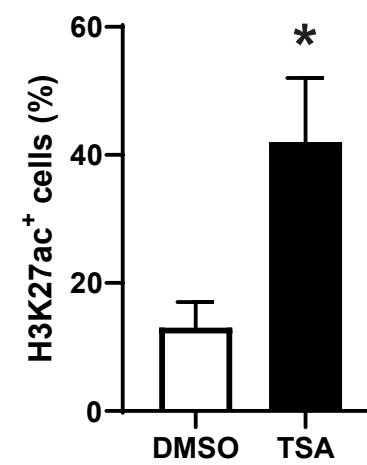

Supplement: Supplementary file 14 — Additional file 14: Figure S8. (A) Representative images of immunofluorescence staining of the H3K27ac distribution in the female left gonads treated with TSA or DMSO. Note the TSA treated female right gonad showed increased and punctate H3K27ac signal compared to the DMSO. Scale bar: 20 μm. (B) The quantifications of the H3K27ac positive nuclei in the gonads of different treatments. Data was mean ± SEM, n = 4, * p < 0.05 by Student t test. [file 13293_2022_415_MOESM14_ESM.pdf]
