# Supplementary material for: H3K27ac chromatin acetylation and gene expression analysis reveal sex- and situs-related differences in developing chicken gonads
Source: Biol Sex Differ. 2022 Feb 8;13:6. doi: 10.1186/s13293-022-00415-5 (PMC8822763; doi:10.1186/s13293-022-00415-5)
Supplement: Supplementary file 9 — Additional file 9: Figure S3. Correlation test of ChIP-Seq data. (A-D) Correlation test between the biological replicates within the same gonad. (E–H) Correlation test between the combined data and inputs from the same gonad. Note that the correlation between biological repeats is high and the correlation between IP and input is low. [file 13293_2022_415_MOESM9_ESM.pdf]

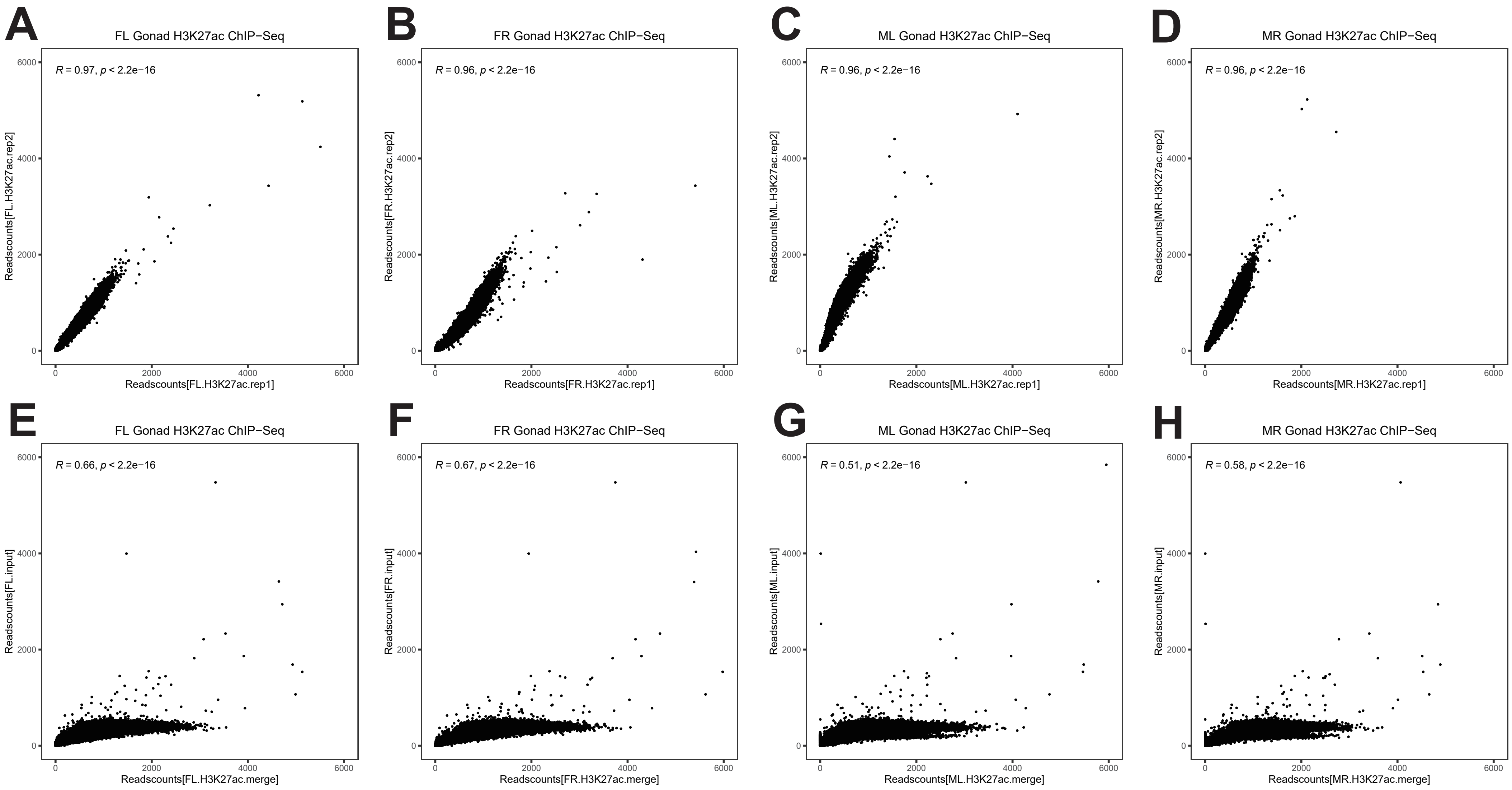

A
FL Gonad H3K27ac ChIP−Seq
R = 0.97, p < 2.2e−16
Readscounts[FL.H3K27ac.rep2]
Readscounts[FL.H3K27ac.rep1]
B
FR Gonad H3K27ac ChIP−Seq
R = 0.96, p < 2.2e−16
Readscounts[FR.H3K27ac.rep2]
Readscounts[FR.H3K27ac.rep1]
C
ML Gonad H3K27ac ChIP−Seq
R = 0.96, p < 2.2e−16
Readscounts[ML.H3K27ac.rep2]
Readscounts[ML.H3K27ac.rep1]
D
MR Gonad H3K27ac ChIP−Seq
R = 0.96, p < 2.2e−16
Readscounts[MR.H3K27ac.rep2]
Readscounts[MR.H3K27ac.rep1]
E
FL Gonad H3K27ac ChIP−Seq
R = 0.66, p < 2.2e−16
Readscounts[FL.input]
Readscounts[FL.H3K27ac.merge]
F
FR Gonad H3K27ac ChIP−Seq
R = 0.67, p < 2.2e−16
Readscounts[FR.input]
Readscounts[FR.H3K27ac.merge]
G
ML Gonad H3K27ac ChIP−Seq
R = 0.51, p < 2.2e−16
Readscounts[ML.input]
Readscounts[ML.H3K27ac.merge]
H
MR Gonad H3K27ac ChIP−Seq
R = 0.58, p < 2.2e−16
Readscounts[MR.input]
Readscounts[MR.H3K27ac.merge]
0
2000
4000
6000
